# LESO: A ten-year ensemble of satellite-derived intercontinental hourly surface ozone concentrations

**DOI:** 10.1038/s41597-023-02656-4

**Published:** 2023-10-25

**Authors:** Songyan Zhu, Jian Xu, Jingya Zeng, Chao Yu, Yapeng Wang, Haolin Wang, Jiancheng Shi

**Affiliations:** 1 National Space Science Center, Chinese Academy of Sciences, Beijing, 100190 China; 2 School of GeoSciences, National Center for Earth Observations, University of Edinburgh, Edinburgh, EH9 3FF UK; 3 Department of Economics, Business School, University of Exeter, Exeter, EX4 4PU UK; 4 Aerospace Information Research Institute, Chinese Academy of Sciences, Beijing, 100094 China; 5 Key Laboratory of Radiometric Calibration and Validation for Environmental Satellites, National Satellite Meteorological Center, China Meteorological Administration, Beijing, 100081 China

**Keywords:** Atmospheric science, Environmental sciences

## Abstract

This study presents a novel ensemble of surface ozone (O_3_) generated by the LEarning Surface Ozone (LESO) framework. The aim of this study is to investigate the spatial and temporal variation of surface O_3_. The LESO ensemble provides unique and accurate hourly (daily/monthly/yearly as needed) O_3_ surface concentrations on a fine spatial resolution of 0.1◦ × 0.1◦ across China, Europe, and the United States over a period of 10 years (2012–2021). The LESO ensemble was generated by establishing the relationship between surface O_3_ and satellite-derived O_3_ total columns together with high-resolution meteorological reanalysis data. This breakthrough overcomes the challenge of retrieving O_3_ in the lower atmosphere from satellite signals. A comprehensive validation indicated that the LESO datasets explained approximately 80% of the hourly variability of O_3_, with a root mean squared error of 19.63 *μ*g/m^3^. The datasets convincingly captured the diurnal cycles, weekend effects, seasonality, and interannual variability, which can be valuable for research and applications related to atmospheric and climate sciences.

## Background & Summary

Surface ozone (O_3_) pollution is a global concern due to its detrimental effects on public health^1^ and food security^2^. Surface ozone (O_3_), also known as ground-level O_3_ (up to roughly 3 km above the Earth’s surface), is formed through chemical reactions in the troposphere between volatile organic compounds (VOCs) and nitrogen oxides (NO_x_) in the presence of sunlight^3^. According to the latest global air quality guidelines (AQG-2021^4^), the recommended level for the average of daily maximum 8-hour mean O_3_ concentration is 100 *μ*g/m^3^. Long-term exposure to elevated levels of O_3_ has been found to result in the development of cardiovascular and respiratory diseases, as well as a decline in lung function^5^. From 2014 to 2021, the daily maximum 8-hour mean O_3_ concentration in Beijing consistently exceeded 100 *μ*g/m^3^ during the months of April to August, with the highest concentration observed in June (~152 *μ*g/m^3^)^6^. In addition, episodes of O_3_ pollution hinder the growth of plants and the accumulation of biomass, consequently leading to a decrease in crop yield^7,8^. Meanwhile, the connection between surface O_3_ and climate change has garnered considerable attention in academic discourse^9–11^.

The community has made significant progress in estimating regional surface O_3_ concentrations by integrating ground-based site measurements with satellite remote sensing^12,13^. However, the majority of these studies have focused on the daily surface O_3_ levels over China. To better analyze the spatial and temporal variability of surface O_3_ on a broader scope, it is valuable to generate a comprehensive ensemble of surface O_3_ concentrations that encompasses various hotspot regions worldwide. These datasets will not only contribute to an enhanced understanding of ecosystem resilience to climate change but also provide recommendations for globally coordinated O_3_ regulation.

The LEarning Surface Ozone (LESO)^6,14^ is a subset of the Learning Air Pollutants from Satellite Observations (LAPSO)^15^ system that employs advanced deep learning techniques to integrate multi-source datasets and infer spatial and temporal variability of air pollutants. The primary objective of LESO is to improve our understanding of the interactions between the atmospheric environment and human activities^6^. We used the state-of-the-art deep forest method^16,17^ to establish a relationship between the ground-based O_3_ measurements and satellite observations, as well as meteorological reanalysis records. The deep forest method was suggested because it yielded more accurate estimation of O_3_ concentrations, with an approximate increase of 30% in accuracy, as compared to conventional machine learning techniques such as shallow-layer neural networks and decision trees (e.g., multiple layer perceptron and random forest)^14^. The trained functions between the input variables (satellite observations and meteorological parameters) and output variables (surface O_3_ measurements) were subsequently applied to produce gridded estimates of O_3_. As most abundances are concentrated in the stratosphere, the signal of O_3_ in the lower troposphere observed by nadir-viewing satellites is rather weak^18,19^. A comprehensive analysis using multiple satellite data sources has indicated that the application of deep learning techniques can achieve reliable and consistent estimation outcomes^14^. This capability enables the utilization of the vast potential of existing satellite data to derive surface ozone with high resolution and extensive coverage.

For this purpose, we adopted the LESO estimation framework to generate surface O_3_ data products for a period of 10 years (2012–2021) in three regions: the Chinese mainland (abbreviated as “China” hereafter), Europe, and the United States (US), including nearly 30 countries in total. The data was obtained at hourly temporal and 0.1° × 0.1° spatial resolutions. The LESO ensemble possesses the capability to investigate the long-term spatiotemporal characteristics of surface O_3_ concentrations across a wider geographical range than any other currently available datasets. In addition to the statistical validation, the LESO surface O_3_ datasets were assessed in four scenarios:

- O_3_ variability during rush hours: O_3_ in the troposphere is formed by the photochemical reaction involving nitrogen oxides (NO_x_) that are commonly emitted from combustion exhaust^20^.
- O_3_ weekend effect^21^: Higher O_3_ concentrations are typically observed on weekends in urban areas^22^.
- O_3_ seasonality: O_3_ pollution events tend to occur in spring and summer when the solar radiation is strong^23^.
- O_3_ interannual variability: This can be a result of regulatory policies and/or major social incidents, e.g., the implementation of lockdown measures during the COVID-19 pandemic^24^.

## Methods

### Deep-learning model training and validation

**Fig. 1:**
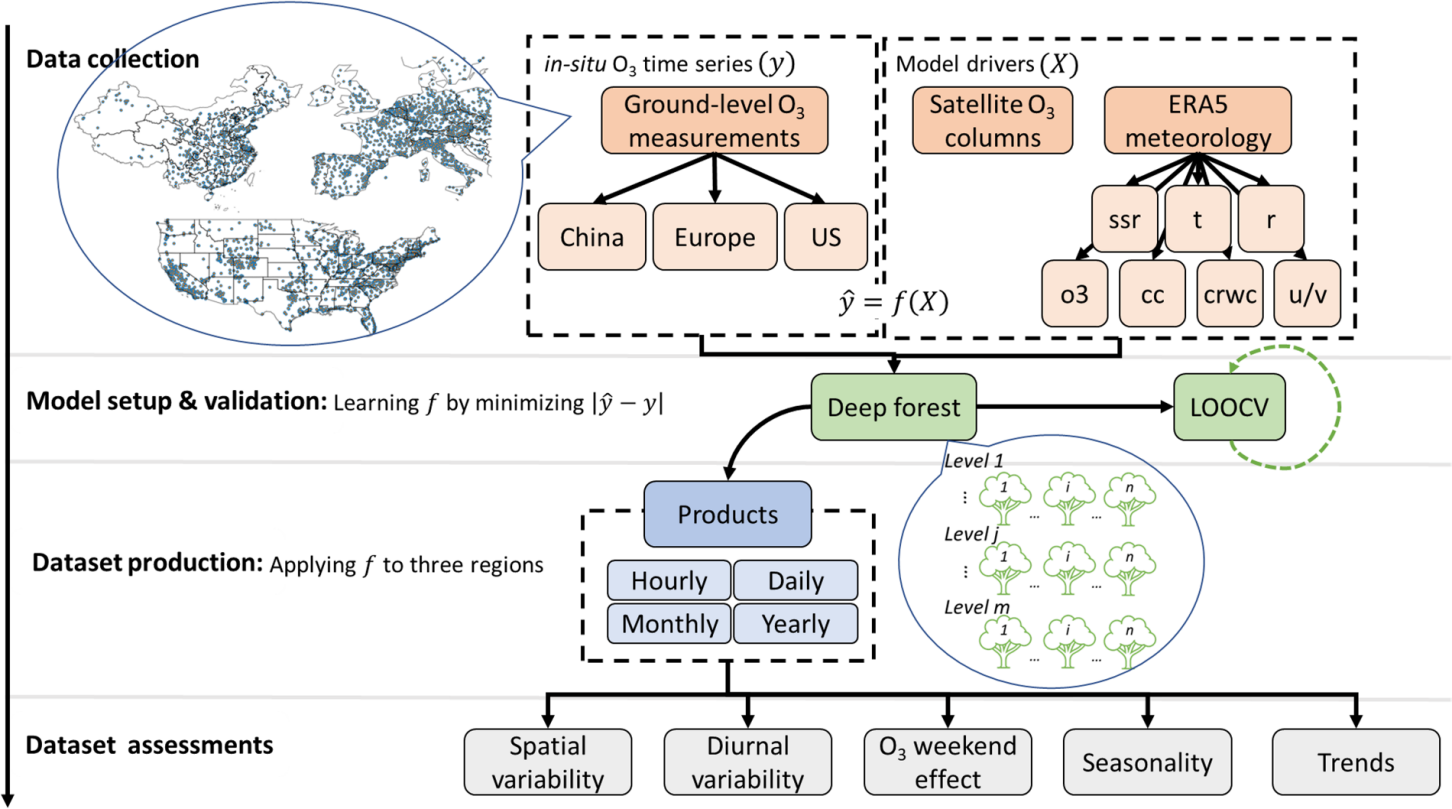
The workflow of generating and validating of the LESO ensemble. The main procedures include data collection, model setup and validation, dataset production, and dataset assessments. The region “China” represents the geographical area of the mainland. The region “Europe” includes 25 countries, i.e., Albania, Andorra, Austria, Belgium, Bosnia and Herzegovina, Croatia, Czechia, Denmark, France, Germany, Hungary, Ireland, Italy, Luxembourg, Montenegro, Netherlands, Norway, Poland, Portugal, Slovakia, Slovenia, Spain, Sweden, Switzerland, and the United Kingdom of Great Britain and Northern Ireland. The region “US” refers to the continental United States. We used seven meteorological variables: shortwave solar radiation (ssr) and vertical profiles of temperature (t), O_3_ mixing ratio (o3), relative humidity (r), U-/V- wind components (u/v), cloud cover (cc), and rain water content (crwc) at 200 hPa, 500 hPa, 700 hPa, 900 hPa, and 1000 hPa. The term “LOOCV” refers to the Leave-One-Out Cross-Validation.

Figure 1 illustrates the main procedures involved in generating and validating the LESO ensemble. The generation of the LESO ensemble relies on deep learning algorithms extracting the nonlinear relationship between surface O_3_ measurements obtained from *in-situ* environmental monitoring sites and the corresponding satellite/climate data at the same location. The workflow consists of four major steps: data collection, model setup and validation, dataset production, and assessments. The deep learning method considered in this study is the DF21 (Deep Forest v2021.2.1^17^) model, which is characterized by its cascading decision forests structure (refer to Fig. 1). The DF21 model was trained and validated using *in-situ* O_3_ measurements obtained from local environmental agencies in the three regions (China, Europe and the US). The independent variables driving the DF21 model, as shown in Fig. 1, are the satellite-derived O_3_ total columns obtained from the Ozone Monitoring Instrument (OMI)^25^ and the meteorological parameters derived from the fifth-generation European Centre for Medium-Range Weather Forecasts (ECMWF) atmospheric reanalysis of the global climate (ERA5)^26^. The ERA5 meteorological parameters included shortwave solar radiation, vertical profiles of temperature, relative humidity, wind, U-/V- wind components, rain water content, and O_3_ mixing ratio (see Fig. 1). The quality of the corresponding data has been evaluated through the use of independent *in-situ*/satellite-based datasets and global models^27^. The satellite-derived total columns provide an overview of spatial distribution of in the atmosphere, whereas the ERA5 data products enhance our understanding of the impact of meteorological conditions on the physio-chemical processes involved in the formation and behavior of atmospheric O_3_. In addition, we have analyzed the estimation performance using data from the TROPOspheric Monitoring Instrument (TROPOMI) on board the Sentinel-5P satellite, which serves as an independent verification. Further details can be found in the subsequent section. We utilized a total of 4821 *in-situ* environmental monitoring sites, comprising 1628 sites from the China National Environmental Monitoring Center (CNEMC), 1866 sites from the European Environmental Agency (EEA), and 1327 sites from the Environmental Protection Agency (EPA). The maintenance of data quality for these *in-situ* measurements is the responsibility of the respective data provider. Please refer to the provided links for more information:

- OMI O_3_ data^28^: https://acdisc.gesdisc.eosdis.nasa.gov/data/Aura_OMI_Level3/OMDOAO3e.003/.
- TROPOMI O_3_ data^29^: https://developers.google.com/earth-engine/datasets/catalog/COPERNICUS_S5P_NRTI_L3_O3.
- ERA5 global meteorological reanalysis^30^: 10.24381/cds.143582cf.
- *in-situ* measurements in China (CNEMC)^31^: https://air.cnemc.cn:18007.
- *in-situ* measurements in Europe (EEA)^32^: https://www.eea.europa.eu/themes/air/explore-air-pollution-data.
- *in-situ* measurements in the US (EPA)^33^: https://www.epa.gov/outdoor-air-quality-data.

In this study, the TROPOMI O_3_ total columns were taken from the near-real-time (NRTI) product, which has a high level of data quality and demonstrates consistent reliability when compared to the offline (OFFL) product^34,35^. The prompt availability of the NRTI O3 product further highlights its notable advantage in terms of timeliness. It is important to acknowledge that the training process of the DF21 model requires the optimization of fine-tuning parameters. For more detailed information, please refer to the corresponding documents^6,15,17^.

The DF21 model was validated for all *in-situ* sites using the Leave-One-Out Cross-Validation (LOOCV) approach that is reliable when dealing with small datasets^36^. A total of 4821 validations were conducted for the DF21 model. In each validation, the data from one site was used as the training dataset, while the data from all other sites were used as the test dataset. The gridded feature data were interpolated at the site level using the inverse distance weighting method^37^. To synchronize with the *in-situ* measurements and ERA5 meteorological data, the daily satellite O_3_ total columns were linearly interpolated to the hourly timescale in the temporal dimension. The interpolation process involved the identification of “good” pixels based on QA flags and a cloud fraction below 10%. The LESO framework distinguishes itself from other data-driven estimation methods^12,13,38^ by adopting the dynamical networking technique^6^, which incorporates data from nearby sites to train the model. This technique makes it possible to mitigate the effects of uncertainties arising from factors like topography and regional climatic conditions^39,40^. To assess the performance of the validation, we used the coefficient of determination (R^2^), root mean squared error (RMSE), mean absolute error (MAE), and mean bias error (MBE).

### Surface O_3_ datasets production and assessment

The trained DF21 model was employed to generate datasets of intercontinental surface O_3_. The production process entailed incorporating the model with 10-year gridded feature data, including the satellite-derived O_3_ Level-3 total columns data and ERA5 meteorological reanalysis data. The datasets were generated at four distinct temporal resolutions: hourly, daily, monthly, and yearly. The spatial resolution of the datasets was 0.1° × 0.1°, whereas satellite column densities and meteorological reanalysis had a spatial resolution of 0.25° × 0.25°. This improvement in resolution has proven to be feasible through a comparative analysis of O_3_ estimations between OMI and TROPOMI over the period of 2019 to 2021. We noticed that using the utilization of OMI data (0.25° × 0.25°) consistently yielded comparable estimation results when compared to the utilization of TROPOMI data (0.1° × 0.1°). The details of this experiment are presented in Section “Technical Validation”. Besides, our previous work^14,15^ and Figure S4 in “Supplementary Information” have demonstrated that the variability in O_3_ total columns derived from different satellites is deemed to be statistically insignificant. The OMI data seems more advantageous due to the fact that TROPOMI, which was launched in October 2017, only offers Level-3 data products for dates subsequent to late 2018^19,34,35^. Consequently, the spatiotemporal resolution of the LESO datasets can be adjusted to match that of ERA5.

The LESO ensemble was developed based on our previous short-term regional datasets^6,14^ and has undergone substantial revision and enhancement, resulting in datasets that offer a greater level of spatial and temporal detail, spanning a period of more than a decade (not described elsewhere). An extensive validation of the LESO datasets has been conducted from three aspects. Firstly, the spatial distribution of LESO surface O_3_ was compared to ground-level measurements and existing literature. Secondly, the ability of the LESO datasets to accurately replicate widely recognized temporal variation patterns of surface O_3_ was assessed. Lastly, the effectiveness of the LESO datasets in characterizing spatiotemporal distributions of O_3_ was examined. The second and third validations were performed to acquire a deeper insight of the data quality of the LESO ensemble, as it is essential for a reliable model dataset to accurately depict the spatiotemporal variations of O_3_ in the real world. This study focuses on the temporal variation patterns as follows:

- Spatial variability: We analyzed if the LESO datasets can reproduce the elevated O_3_ concentrations during the summer season in eastern China (e.g., the Beijing-Tianjin-Hebei region)^41^, southern Europe (e.g., Spain and Italy)^42^, and the western US (e.g., California)^43^.
- Diurnal variability: We examined the impact of urban road traffic regulations on the nitrogen precursor of O_3_, which is primarily sourced from the transportation sector^44^. The concentration of surface O_3_ is expected to reach its highest level a few hours after the morning rush hour, typically in the mid to late afternoon. This delay is attributed to the time required for photochemical reactions to generate O_3_^45^.
- O_3_ weekend effect: It refers to the phenomenon where the maximum hourly O_3_ levels during weekends can have a decrease of up to 15% compared to weekday levels, or an increase of up to 15%. This effect is believed to be caused by the reduction of nitrogen oxides (NO_x_) in a VOC-limited O_3_ formation regime^46^.
- Seasonality and long-term trends: We analyzed the surface O_3_ variations in response to reduction policies, such as the plan implemented in 2017 by China^47^), as well as the impact of the COVID-19 pandemic since the end of 2019^24,48^.

## Data Records

The LESO ensemble comprises the surface O_3_ datasets over the three regions (see Table 1 for the corresponding geographical range). In the context of this study, the term “China” specifically pertains to the geographical area of the mainland. The term “Europe” stands for the region including a total of 25 countries, namely Albania, Andorra, Austria, Belgium, Bosnia and Herzegovina, Croatia, Czechia, Denmark, France, Germany, Hungary, Ireland, Italy, Luxembourg, Montenegro, Netherlands, Norway, Poland, Portugal, Slovakia, Slovenia, Spain, Sweden, Switzerland, and the United Kingdom of Great Britain and Northern Ireland. The term “US” denotes the geographical area of the continental United States. The datasets were generated at a spatial resolution of 0.1° × 0.1° and across four timescales, i.e., hourly, daily, monthly, and yearly. All of the LESO datasets are available in Zenodo under the Creative Commons Attribution 4.0 International (CC BY 4.0) license.:

- Hourly O_3_ measurements in China^49^: 10.5281/zenodo.7500780.
- Hourly O_3_ measurements in Europe^50^: 10.5281/zenodo.7500782.
- Hourly O_3_ measurements in the US^51^: 10.5281/zenodo.7500784.
- Daily, monthly, and yearly O_3_ measurements in all regions^52^: 10.5281/zenodo.7502204.

**Table 1:** Overview of the LESO satellite-derived surface O_3_ datasets.

	China	Europe	US
Folder Format	<region>-SUR-<pollutant>-<satellite>-<timescale>		
File Format	SUR-<pollutant>-<date>-<model>-<version>		
Latitude Range	5.01° N~53.51° N	36.15° N~59.95° N	25.14° N~49.34° N
Longitude Range	73.55° E~134.95°E	10.23° W~19.87°E	124.64° W~67.04° W
Spatial Resolution	0.1° × 0.1°	0.1° × 0.1°	0.1° × 0.1°
Dataset Size (Hourly)	154.00 GB	46.50 GB	91.10 GB
Dataset Size (Daily)	6.43 GB	1.93 GB	3.79 GB
Dataset Size (Monthly)	217.00 MB	66.10 MB	128.00 MB
Dataset Size (Yearly)	18.10 MB	5.51 MB	10.70 MB

The data files are organized based on region and timescale using the Network Common Data Form, version 4 (NetCDF-4) format, following the naming convention outlined in Table 1. As an example, a file named “SUR-O3-2012-01-03-DF21-01.nc” in the “EU-SUR-O3-OMI-Hourly” directory stores the hourly measurements of surface O_3_ concentrations (version 01) on January 3, 2012 derived from the OMI instrument using the DF21 model (https://deep-forest.readthedocs.io/en/stable/). The open access in-built processing tool allows for dynamical of the estimation uncertainty at user-defined geolocations^53^. To spatially extrapolate the site-level uncertainties to the regions of interest, we employed a geographically weighted regression technique.

## Technical Validation

### Statistical validation

**Fig. 2:**
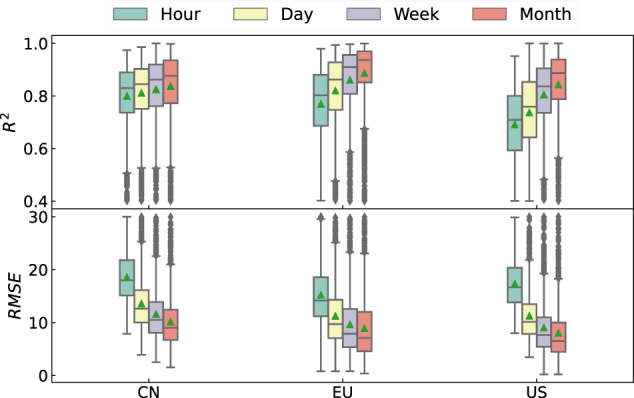
Site-level validation statistics of the LESO ensemble in terms of boxplots in all the three regions for four timescales (hourly, daily, weekly, and monthly). The unit of RMSE is *μ*g/m^3^. The green triangles in the boxes are the mean values of the coefficient of determination (R^2^) and root mean squared error (RMSE).

The LOOCV results for the LESO ensemble, using a total of 4821 *in-situ* sites, demonstrated excellent performance. Please refer to Fig. 2 and Table 2 for a summary. The mean values of R^2^ and RMSE were 0.78 and 12.84 *μ*g/m^3^, respectively, indicating a strong correlation and relatively small deviation between the predicted and observed measurements. The average site-level concentration of surface O_3_ in China, Europe, and the US during the summer months was 75, 67, and 65 *μ*g/m^3^, respectively. In contrast, during the winter months, the average concentrations were 40, 39, and 51 *μ*g/m^3^ in China, Europe, and the US, respectively. From the hourly timescale to the monthly timescale, the R^2^ values for the first quartile ranged from 0.67 to 0.80, while the RMSE for the third quartile ranged from 11.71 to 20.30 *μ*g/m^3^. As expected, the validation results were superior at coarser timescales compared to finer timescales, supporting by a higher level of explained O_3_ variation (R^2^) and a lower magnitude of estimation errors (RMSE). The R^2^ value for the hourly timescale (~0.76) was found to be lower than those for the daily, weekly, and monthly timescales by 4.44%, 8.91%, and 11.52%, respectively. The RMSE for the hourly timescale (~16.93 *μ*g/m^3^) was observed to be higher than those for the daily, weekly, and monthly timescales by 40.93% 67.16% and 86.19%, respectively. The interquartile range (IQR) values of R^2^ for the hourly, daily, weekly, and monthly timescales were 0.20, 0.19, 0.17, and 0.15, respectively. The IQR values of RMSE for the hourly, daily, weekly, and monthly timescales were 7.19, 6.71, 6.69, and 6.52 *μ*g/m^3^, respectively.

**Table 2:** Validation metrics for hourly LESO O_3_ measurements in the three regions.

	R^2^	RMSE	MBE	MAE	Slope	Intercept	P val.	Std. Err.
	China							
Min	0.40	7.88	−43.23	5.36	0.16	−23.10	0.00	0.00
Q1	0.74	15.12	−3.62	10.33	0.77	2.79	0.00	0.00
Median	0.83	17.99	−0.65	12.47	0.87	6.80	0.00	0.00
Mean	0.80	18.56	−0.94	13.60	0.84	8.69	0.00	0.00
Q3	0.89	21.82	2.61	15.80	0.94	12.96	0.00	0.00
Max	0.97	30.00	30.86	51.92	1.14	60.50	0.00	0.02
	Europe							
Min	0.40	0.80	−54.18	0.80	0.05	−63.27	0.00	0.00
Q1	0.69	11.20	−5.15	8.33	0.71	2.08	0.00	0.00
Median	0.80	14.17	−0.15	10.89	0.83	7.73	0.00	0.00
Mean	0.77	15.24	−0.91	12.78	0.79	10.58	0.00	0.00
Q3	0.88	18.56	4.38	14.98	0.90	16.86	0.00	0.00
Max	0.98	30.00	53.87	54.78	1.37	78.78	0.82	1.43
	US							
Min	0.40	7.99	−38.85	5.79	0.06	−27.20	0.00	1.70
Q1	0.59	13.81	−3.51	10.31	0.49	10.64	0.00	3.42
Median	0.71	16.66	0.53	12.71	0.65	21.16	0.00	4.33
Mean	0.69	17.35	0.26	13.80	0.64	22.70	0.00	6.04
Q3	0.80	20.36	4.66	16.10	0.79	32.39	0.00	7.15
Max	0.95	29.88	34.57	40.11	1.15	89.55	0.00	141.95

The Q1 and Q3 represent the first and third quartiles, respectively. The MBE and MAE represent the mean bias and mean absolute errors, respectively. The terms “P val.” and “Std. Err.” stand for the *p*-value and standard error, respectively.

The validation results showed a similar level of performance (R^2^ and RMSE) between China and Europe, but the results in the US were slightly inferior, particularly when considering the hourly timescale. On average, across the four timescales, the mean and IQR of R^2^ were 0.82 and 0.16 in China, 0.83 and 0.18 in Europe, and 0.77 and 0.22 in the US. The mean and IQR values of RMSE in China were 13.39 and 8.16 *μ*g/m^3^, respectively. In Europe, the mean and IQR values were 11.24 and 8.40 *μ*g/m^3^, respectively, while in the US, they were 11.38 and 8.76 *μ*g/m^3^, respectively. The R^2^ in the US was 7% lower than that in China and Europe. However, the corresponding RMSE in the US was 15% lower than that in China. In China, it was observed that there was a positive correlation between the R^2^ and RMSE, while in the US, the opposite trend was observed. Factors causing this correlation might be in relation to the distribution of *in-situ* sites and the O_3_ formation mechanism. The average O_3_ concentration over multiple years in China, Europe, and the US was 60, 53, and 61 *μ*g/m^3^, respectively. According to Fig. 1, the *in-situ* sites in China were primarily situated in heavily O_3_-polluted areas (mostly in eastern China)^6,54,55^, whereas many sites in the US were located in regions with low O_3_ levels, such as the east coastal area^56^. In addition, the O_3_ formation pathway varies significantly between the eastern and western areas of the US^14^. The notable difference in O_3_ concentration levels between the eastern and western regions of the US can be attributed to the transport of O_3_ from the stratosphere to troposphere, a phenomenon known as the stratospheric intrusions^57^. The intrusions usually occur in relatively high-latitude areas like the western part of the US^58^. In contrast, the O_3_ formation pathway in China was rather consistent between areas^59^. This may explain why LESO produced lower R^2^ and lower RMSE in the US. The validation outcome for hourly surface O_3_ formation in the US was arguably promising, considering its complexity. Nevertheless, further validations are required to analyze the spatiotemporal variation characteristics for justifying the long-term reliability of LESO.

### Validation of temporal variability

**Fig. 3:**
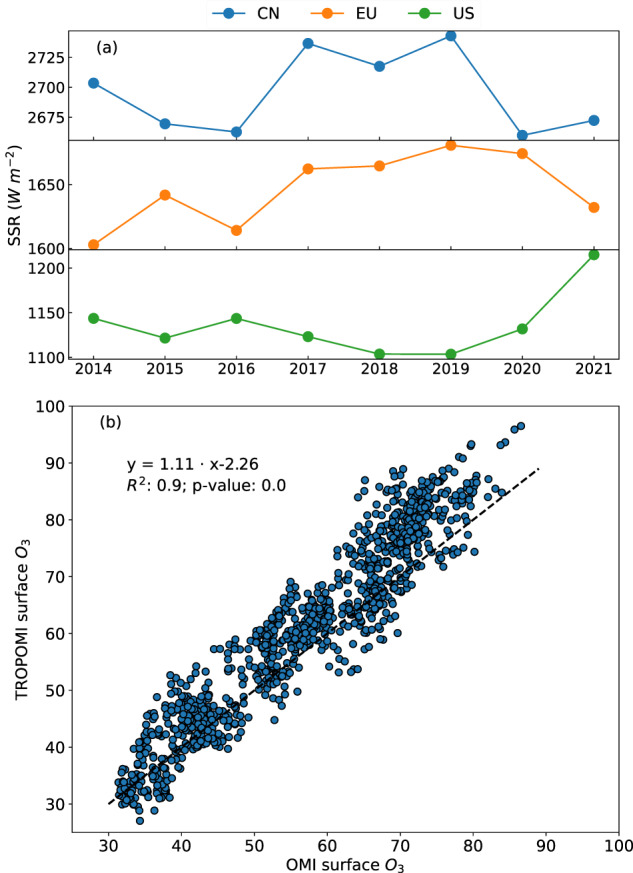
(**a**) Yearly averaged solar shortwave radiation (SSR) from the ERA5 reanalysis in all the three regions. (**b**) Scatter plot of estimated surface O_3_ concentrations (*μ*g/m^3^) derived from OMI against those derived from TROPOMI in all the three regions between 2019 and 2021.

Tropospheric O_3_ is a secondary air pollutant formed from photochemical reactions^60^:

1 $${\rm{V}}{\rm{O}}{\rm{C}}{\rm{s}}+{\rm{N}}{{\rm{O}}}_{{\rm{x}}}\mathop{\to }\limits^{h\nu (\lambda  < 420{\rm{n}}{\rm{m}})}{{\rm{O}}}_{3},$$

where VOCs and NO_x_ refer to volatile organic compounds and nitrogen oxides, respectively, *hv* represents the strength of SSR. Figure 3a demonstrates that the three regions experienced varied interannual solar shortwave radiation (SSR) from 2014 to 2021, which can be associated with the variability of surface O_3_^61–63^. In China, the SSR difference between 2014 and 2021 was smaller than 100 Wm^−2^, and higher SSR values were found during 2017–2019. In Europe, the SSR exhibited an overall upward trend, and the SSR difference between these years was also smaller than 100 Wm^−2^. In the US, the SSR difference during the same period reached 110 Wm^−2^, and an overall downward trend was seen from 2014 to 2019. The SSR in the US increased rapidly since 2019, particularly reaching 1214 Wm^−2^ in 2021. Figure 3b compares the LESO ensemble of surface O_3_ between 2019 and 2021 in the three regions at a spatial resolution of 0.1° × 0.1° using the two products of total O_3_ from OMI and TROPOMI, respectively. The R^2^ between the OMI-based and TROPOMI-based surface O_3_ concentrations was 0.9, and the slope of linear regression was 1.1. The surface O_3_ estimates based on TROPOMI were 10% higher than those based on OMI. Providing higher spatial resolution and longer operational period, the next version of LESO will consider the TROPOMI O_3_ data for future long-term use.

**Fig. 4:**
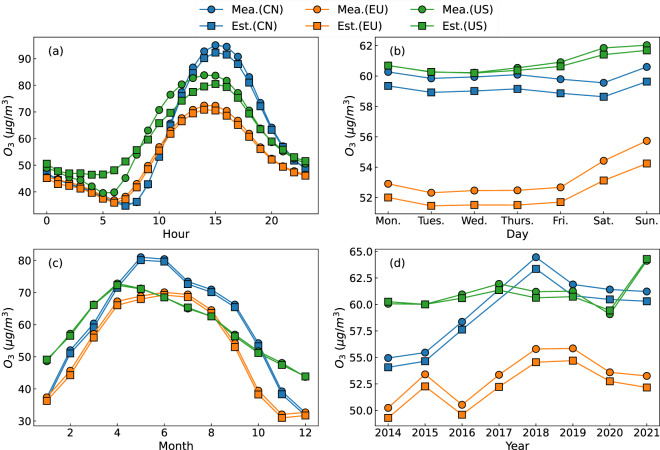
Temporal variability of surface O_3_ at the hourly (**a**), daily (**b**), monthly (**c**), and yearly (**d**) scales. The curves are plotted using the *in-situ* measurements (“Mea.”) and LESO ensemble (“Est.”).

The LESO ensemble was validated by analyzing the temporal variation characteristics of surface O_3_ at four time scales: diurnal cycles (Fig. 4a), weekend effect (Fig. 4b), seasonality (Fig. 4c), and interannual variations (Fig. 4d). The estimated surface O_3_ concentrations were consistent with the ground-based measurements with a mean difference of less than 1 *μ*g/m^3^. Both datasets showed strong diurnal patterns caused by urban commutes^64^: the peak values were observed at 3 PM, while the trough values occurred between 6 to 9 AM. The same findings have bee confirmed by the relevant literature^65,66^. The LESO ensemble accurately reproduced the peak and trough values of surface O_3_ in the regions of China and Europe, but slightly overestimated the values in the early morning and underestimated them at noon in the US.

The estimated and measured surface O_3_ showed significant weekend effects in all three regions, with higher O_3_ values on weekends compared to weekdays (see Fig. 4b), which has been discussed in previous studies^22,44,46^. The estimated daily average O_3_ values were slightly smaller than the ground-based measurements. The underestimation in China and Europe was approximately 1 *μ*g/m^3^, whereas in the US it was less than 0.05 *μ*g/m^3^. The stronger weekend effects were seen in Europe (~2.51 *μ*g/m^3^) than in China (~0.09 *μ*g/m^3^) and the US (~1.41 *μ*g/m^3^).

The existing literature^20,67^ suggests that higher surface O_3_ concentrations were seen in summer than in winter. This seasonal variability of surface O_3_ was identified in the three regions from both the LESO datasets and ground-based measurements. The highest O_3_ concentration in China, Europe, and the US occurred in April, May, and June, respectively. The monthly difference between the measured and estimated O_3_ was less than 1 *μ*g/m^3^ in the three regions.

Figure 4d confirms that the LESO datasets can reconstruct realistic interannual variations of O_3_. The difference between the yearly measured and estimated O_3_ levels in China and Europe was 0.92 and 1.06 *μ*g/m^3^, respectively, whereas it was only 0.17 *μ*g/m^3^ in the US. The existing literature^54,68^ has highlighted an increasing trend in surface O_3_ over China, which can be attributed to the rapid urbanization and industrialization progress (e.g., increased combustion and industrial pollutant emissions). As illustrated in Fig. 4d, surface O_3_ concentrations in China experienced a rapid increase from 2014 to 2018, with an annual growth rate of 3.12 *μ*g/m^3^. However, between 2018 and 2021, there was a downward trend in O_3_ concentrations, which was likely due to the implementation of regulations by the authorities to address air pollution issues^41,69,70^, as well as the lockdowns imposed during the COVID-19 pandemic^71–74^. O_3_ concentrations in Europe exhibited an overall increasing trend, with an annual growth rate of 0.48 *μ*g/m^3^. These interannual variation patterns of O_3_ were consistent with those of SSR shown in Fig. 3a. Because Europe has not implemented stringent measures to reduce O_3_ pollution since the Gothenburg Protocol in 2012^75^, the intensity of solar radiation can play a significant role in interacting with O_3_ variations^20^. Figure 4d shows that the time series of O_3_ in the US was generally stable, except for the sudden change in 2021. Likewise, the relevant authority in the US has not issued any other comprehensive reduction plan apart from the Clean Air Act Amendments of 1990^76^. The variations of SSR (see Fig. 3a) may largely contribute to the observed trend of surface O_3_ in the US.

### Validation of spatiotemporal distribution

**Fig. 5:**
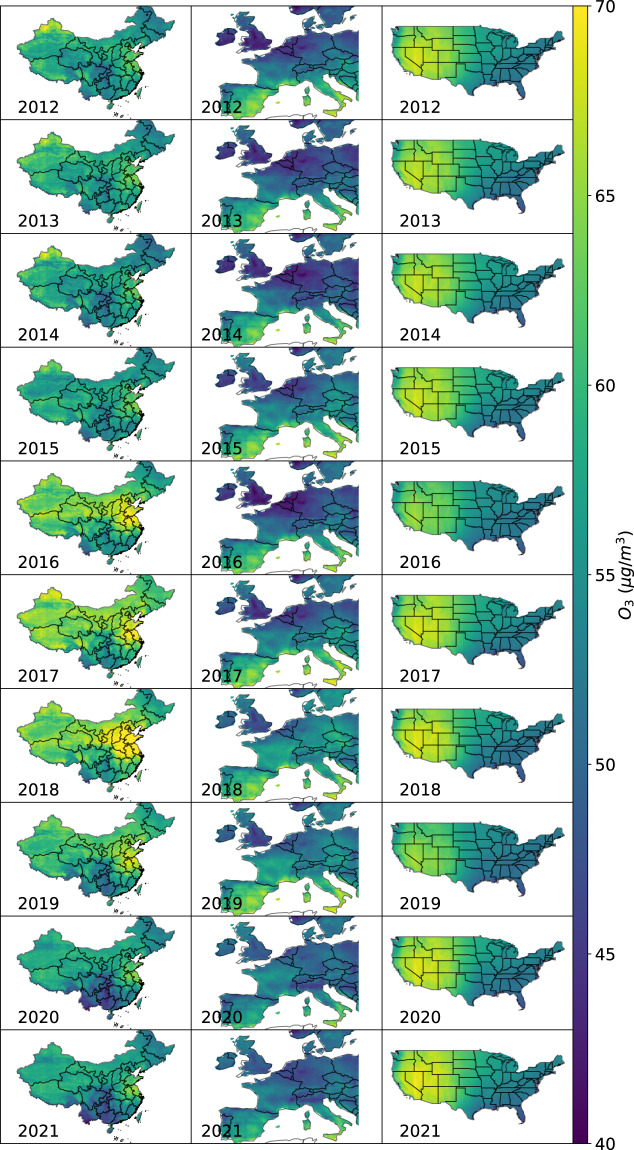
Spatial variations of surface O_3_ plotted with the LESO ensemble from 2012 to 2021 in China (left column), Europe (middle column), and the US (right column).

Figure 5 illustrates the yearly mean of surface O_3_ from the LESO ensemble in the three regions from 2012 to 2021. In line with the previous studies^77–79^, high levels of O_3_ were found in the North China Plain, also known as “Jing-Jin-Ji” area. O_3_ concentrations in Europe were low in both spatial and temporal domains. As compared to northern Europe, severe O_3_ pollution was found in southern Europe, which can be caused by the latitudinal distribution of solar radiation^20,80–82^. In the US, O_3_ concentrations were generally stable, with relatively low levels observed between 2016 and 2019. The spatial distribution of O_3_ differed considerably between the western and eastern parts of the US. Specifically, the western part consistently exhibited higher O_3_ concentrations than the eastern part throughout the years. This spatial discrepancy agrees with the earlier relevant findings^57,58^ and may be a result of the stratospheric intrusions. Regarding the difference in interannual variations between the site-level estimates (see Fig. 4d), the possible factors are: (1) the number of sites varied throughout the years, and (2) the sites were located mainly in highly polluted areas^6,14^. The spatial distribution of O_3_ in China, as characterized by the LESO ensemble, agrees with the findings of recent studies^83,84^. Unfortunately, there are no other available data products for validating the LESO ensemble in Europe and the US.

Furthermore, the LESO ensemble was validated with the GEOS-Chem (GEOS: Goddard Earth Observing System) model^85^, the Community Multiscale Air Quality (CMAQ) model^86^, and the ECMWF Atmospheric Composition Reanalysis 4 (EAC4) model^87^. Both GEOS-Chem and CMAQ models have been extensively applied for simulating air pollutants^88,89^, and the EAC4 model was recently used for validating total ozone columns from TROPOMI^90^. Figure S1 shows that the GEOS-chem model seemed to significantly overestimate surface O_3_ in all the three regions, whereas the EAC4 model yielded lower estimates than the LESO ensemble. The LESO, CMAQ, and EAC4 model datasets exhibited similar spatial patterns of O_3_ in the US, and the CMAQ model captured more detailed spatial features in the western region of the US. The comparison between LESO, CMAQ, and EAC4 (see Figure S2) confirmed underestimated O_3_ concentrations found by the EAC4 model. Figure S3 shows the site-level validations of the EAC4 and CMAQ models using ground-based measurements. The median R^2^ values of the EAC4 model in all the regions were below 0.6, while the median R^2^ of the CMAQ model in the US was about 0.45. The validation results of the CMAQ and EAC4 models appeared to be worse than those of LESO (see Fig. 2). Due to the scope of this study, readers are kindly directed to “Supplementary Information” for a detailed elaboration.

## Usage Notes

The LESO surface O_3_ datasets were generated separately for four timescales and three regions. As the data size varies greatly across different timescales, such as from 154 GB for the hourly timescale to 18.1 MB for the yearly timescale in China, we recommend that users download data based on their specific timescale of interest.

Since O_3_ column data from polar-orbiting satellites are typically provided at a daily or even coarser timescale, it is necessary to temporally interpolate the satellite data in order to generate hourly measurements of surface O_3_. The accuracy of ERA5 hourly meteorological data can be crucial for estimating diurnal variations of surface O_3_. According to the technical validation results, the LESO ensemble can accurately capture the hourly variability of surface O_3_. The LESO O_3_ ensemble in China and Europe has demonstrated greater reliability at the hourly timescale. However, we advise users to exercise caution when using the hourly dataset in the US. The LESO datasets at the other timescales showed similar regional results, indicating no need for additional caution.

The LESO surface O_3_ datasets were produced at the spatial resolution of 0.1° × 0.1°. In case users require datasets with a lower spatial resolution, please contact Songyan Zhu (szhu4@ed.ac.uk) or Jian Xu (xujian@nssc.ac.cn). The current LESO ensemble is publicly available for surface O_3_ measurements in China, Europe, and the US. The authors are also willing to test and apply the LESO framework to other regions of the world, provided that *in-situ* measurements are available.

### Supplementary information

Supplementary Information

## Data Availability

The scripts for processing and reading the LESO datasets are accessible on Github (https://github.com/soonyenju/LESO) under the MIT license. The tools and libraries, including Python v3.9, Numpy v1.20.3, Xarray v0.19.0, Pandas v1.3.3, Deep Forest v2021.2.1 (DF21), scigeo v0.0.13, and sciml v0.0.5, were used to build the LESO framework for generating datasets of surface O_3_ concentrations. The validation of LESO datasets was processed using scitbx v0.0.42 and scikit-learn v0.24.2.
