# Supplementary material for: LESO: A ten-year ensemble of satellite-derived intercontinental hourly surface ozone concentrations
Source: Sci Data. 2023 Oct 25;10:741. doi: 10.1038/s41597-023-02656-4 (PMC10600137; doi:10.1038/s41597-023-02656-4)
Supplement: Supplementary file 1 — Supplementary Information [file 41597_2023_2656_MOESM1_ESM.pdf]

Supplementary Information

Songyan Zhu[1,2,*,†], Jian Xu[1,*,†], Jingya Zeng[3], Chao Yu[4], Yapeng Wang[5], Haolin Wang[2], and Jiancheng Shi[1]

[1]National Space Science Center, Chinese Academy of Sciences, Beijing 100190, China
[2]School of GeoSciences, National Center for Earth Observations, University of Edinburgh, Edinburgh EH9 3FF, UK
[3]Department of Economics, Business School, University of Exeter, Exeter EX4 4PU, UK
[4]Aerospace Information Research Institute, Chinese Academy of Sciences, Beijing 100094, China
[5]Key Laboratory of Radiometric Calibration and Validation for Environmental Satellites, National Satellite Meteorological Center, China Meteorological Administration, Beijing 100081, China
[*]corresponding author(s): Songyan Zhu (szhu4@ed.ac.uk) and Jian Xu (xujian@nssc.ac.cn)
[†]these authors contributed equally to this work

ABSTRACT

This report describes in detail the comparison between the LESO ensemble and two chemical transport models as well as a reanalysis model.

Models

We considered two chemical transport models (CTMs) (GEOS-Chem[1] and CMAQ[2]) and one reanalysis model (EAC4[3]). The GEOS-Chem dataset utilized in this study originated from version 13.3.1[1,4]. The dataset has a spatial resolution of 4° × 5°. The computational time for generating one month of data was about 8 to 9 hours[4]. This comparison considered monthly surface-level data, as it was observed that a finer spatial resolution led to a significant increase in computational time following an exponential trend[4]. The CMAQ dataset was sourced from the United States Environmental Protection Agency's Air QUAlity TimE Series (EQUATES)[5]. We specifically employed the CMAQ daily average surface concentrations dataset including 14 distinct chemical species across the Contiguous US region at a spatial resolution of 12 km. This dataset offers daily average surface information, and each individual file for a month of data occupies approximately 750 MB. Additionally, the CMAQ dataset includes a daily average 3-dimension output for the Northern Hemisphere region (at a spatial resolution of 108 km), specifically designated as "Layer 1 – 44"; however, a single file for a month of data in this configuration is much larger, around 46 GB. Regarding the EAC4 dataset, it features a spatial resolution of 0.75° and a temporal resolution of 3 hours. We selectively utilized the surface-level data corresponding to the atmospheric pressure of 1000 hPa[3].

In light of the extensive amount of modeled data and the unique characteristics of this dataset, we have conducted a thorough analysis that includes examining the spatial distribution, internal consistency, and comparison with *in-situ* measurements for the year 2019.

Results

In relation to spatial distribution in Figure S1, the resolution of the 4° × 5° for the GEOS-Chem model was coarse. Consistent with previous studies[4,6], this model showed clear tendencies of overestimation. Meanwhile, the EAC4 model yielded significantly lower estimates than LESO; however, it exhibited a noticeable level of spatial heterogeneity. In the case of CMAQ, its spatial features closely resembled those of LESO, yet it captured significantly more detailed spatial intricacies compared to LESO. The spatial distribution was generally consistent between these LESO, CMAQ, and EAC4.

Evidenced by the slope of the linear regression analysis Figure S2, it becomes evident that the modeled surface O_3 concentrations from the EAC4 exhibit a notable decrease compared to those from the LESO model, particularly within the geographical context of China. i This discrepancy suggests a potential tendency towards underestimation when considering the LESO results as the benchmark. Specifically, in Europe and the US, the EAC4-modeled O3 concentrations amounted to 56 % of those predicted by the LESO model, whereas in China, this value was reduced to 42 %. Conversely, the level of agreement between the LESO and CMAQ models was conspicuous, devoid of significant instances of either overestimation or underestimation.

In the context of evaluating model O_3 estimates against site measurements, the assessment reveals distinct patterns in Figure S3. The median coefficient of determination (median R^2) showed significant variations across models and regions,

especially when comparing sites within the same geographic region. For instance, in both China and Europe, the median R^2 for the EAC4 model was approximately 0.6, indicating a moderate level of agreement between the model estimates and measurements. In contrast, this value dipped below 0.4 in the US, indicating a relatively weaker agreement between EAC4 estimates and observed values. The median values of the root mean squared error (RMSE) in these three regions were approximately 30 $\mu g/m^3$, 20 $\mu g/m^3$, and 20 $\mu g/m^3$, respectively.

Conversely, the CMAQ model demonstrated better performance in the US. The median R^2 value associated with CMAQ was around 0.45, which exceeded that of EAC4. This indicates a relatively stronger correlation between the CMAQ estimates and actual measurements. Moreover, the median RMSE for CMAQ remained below 15 $\mu g/m^3$, which was notably smaller than the corresponding value for EAC4. It is worth highlighting that the LESO model exhibited exceptional proficiency when compared to *in-situ* measurements.

As indicated by the site-level validations (Figure S4), there was minimal variance in the disparity of R^2 values between the utilization of satellite columns and their absence. The interquartile range measured less than 0.05. Correspondingly, the difference in RMSE was insignificant; both the median RMSE and the interquartile range of RMSE approached negligible values of approximately zero. In line with our previous studies[7,8], the inclusion of satellite-derived data had a limited effect on the accuracy of surface O_3 estimation using machine learning algorithms. This was accomplished by extrapolating measurements from specific monitoring sites.

Discussion

In the broader context, a certain level of coherence can be observed among machine learning, reanalysis, and chemical transport models in general. However, significant disparities become evident when examining their effectiveness in capturing spatial variations of surface O_3, especially when compared to *in-situ* measurements. Employing LESO as the reference standard, the GEOS-Chem model used in this study shows significant overestimations, which aligns with previous research findings. Conversely, outcomes from EAC4 indicate significant underestimations. The CMAQ model, in contrast, aligns more closely with the LESO ensemble. It provides a more intricate depiction, although it still has limitations in accurately capturing variations in accurately capturing variations of O_3 variations when compared to site-level measurements.

This comparison further underscores the inherent limitations of the LESO model. While it agrees with site-level measurements, its representation of spatial variability is still lacking. This shortcoming may be attributed to the exclusive reliance of the LESO model on satellite and meteorological data (Figure S4). Currently, we have included the satellite measurements as model inputs temporarily, with the intention of replacing it with more reliable information in future updates. Enhancing the semi-empirical nature of the LESO model could involve incorporating practical constraints, such as the use of inventory data similar to those employed in CTMs, thereby improving its realism and applicability.

References

1. Bey, I. *et al.* Global modeling of tropospheric chemistry with assimilated meteorology: Model description and evaluation. *J. Geophys. Res. Atmospheres* **106**, 23073–23095 (2001).
2. Appel, K. W. *et al.* The community multiscale air quality (cmaq) model versions 5.3 and 5.3.1: system updates and evaluation. *Geosci. Model. Dev.* **14**, 2867–2897, 10.5194/gmd-14-2867-2021 (2021).
3. Inness, A. *et al.* The cams reanalysis of atmospheric composition. *Atmospheric Chem. Phys.* **19**, 3515–3556 (2019).
4. Wang, H. *et al.* Global tropospheric ozone trends, attributions, and radiative impacts in 1995–2017: an integrated analysis using aircraft (iagos) observations, ozonesonde, and multi-decadal chemical model simulations. *Atmospheric Chem. Phys.* **22**, 13753–13782 (2022).
5. Foley, K. M. *et al.* 2002–2017 anthropogenic emissions data for air quality modeling over the united states. *Data Brief* **47**, 109022 (2023).
6. Eastham, S. D. & Jacob, D. J. Limits on the ability of global eulerian models to resolve intercontinental transport of chemical plumes. *Atmospheric Chem. Phys.* **17**, 2543–2553 (2017).
7. Zhu, S. *et al.* Learning surface ozone from satellite columns (leso): A regional daily estimation framework for surface ozone monitoring in china. *IEEE Transactions on Geosci. Remote. Sens.* **60**, 1–11 (2022).
8. Zhu, S. *et al.* Satellite-derived estimates of surface ozone by leso: Extended application and performance evaluation. *Int. J. Appl. Earth Obs. Geoinformation* **113**, 103008 (2022).

Figures

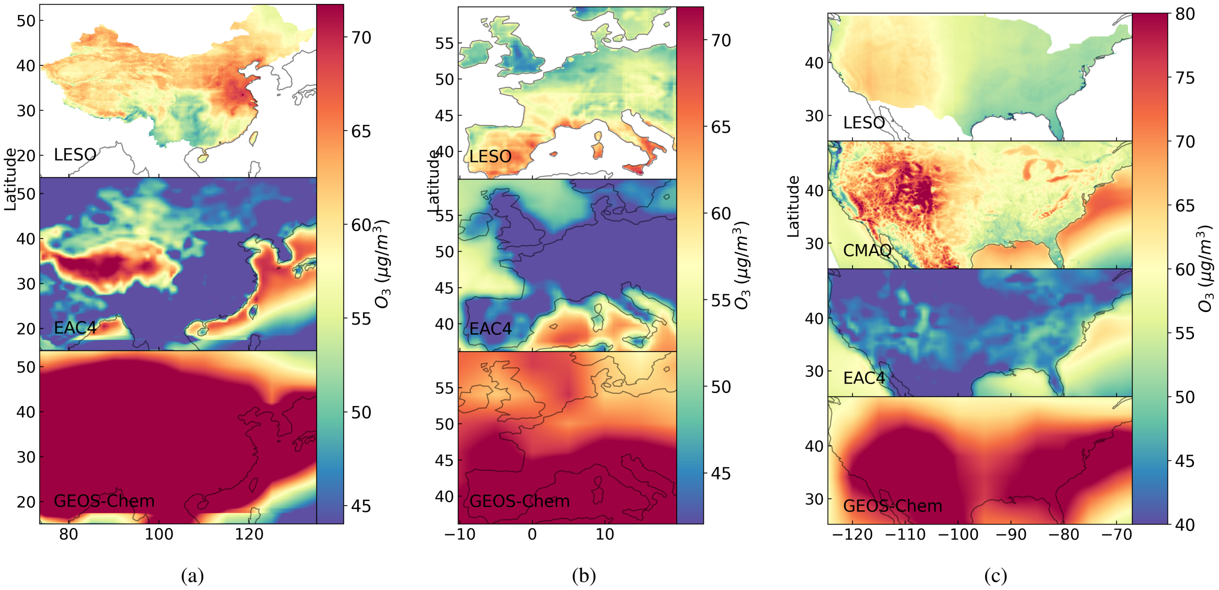

Figure S1. Spatial distributions of surface ozone (O_3) concentrations as simulated by different models in China (a), Europe (b), and the US (c), respectively.

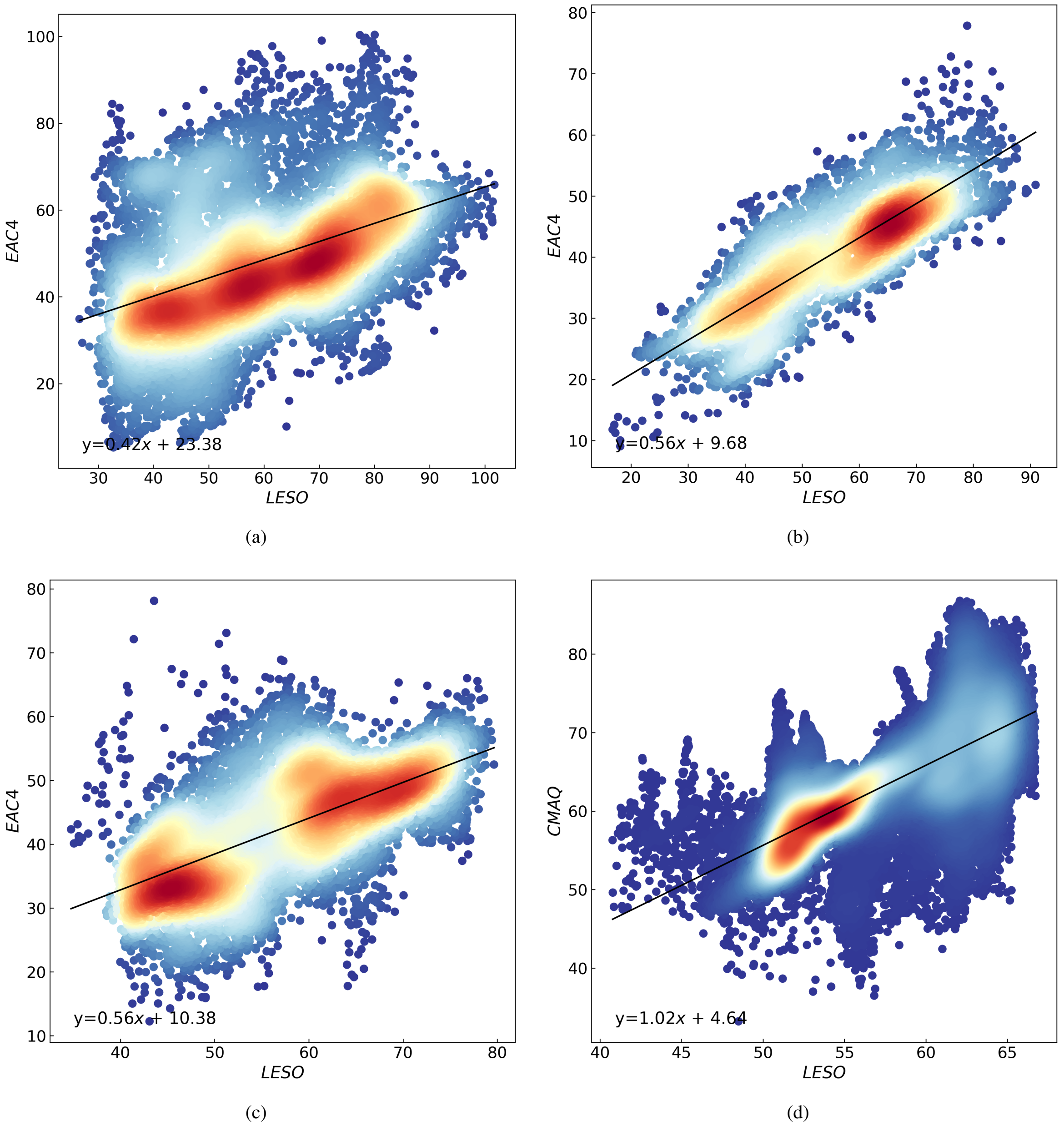

Figure S2. Scatter plots of pixel data from the LESO ensemble compared to the EAC4 pixel data in China (a), Europe (b), and the US (c), respectively, as well as against the CMAQ pixel data specifically in the US (d).

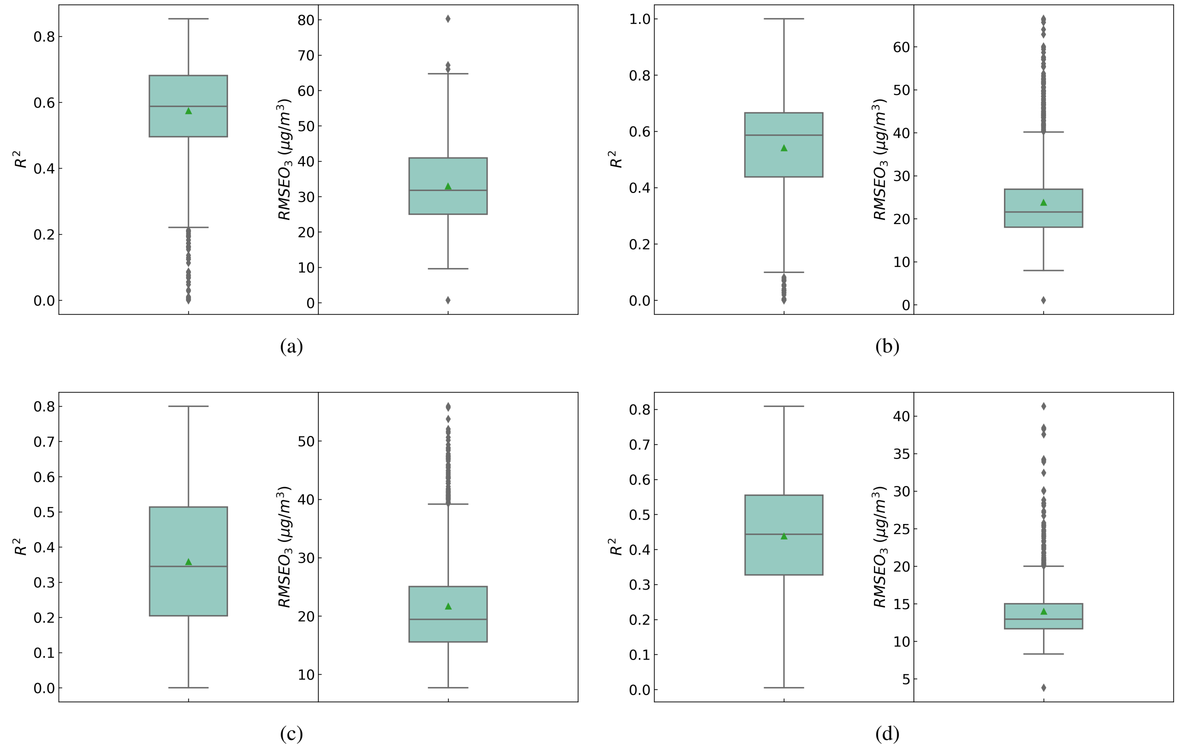

Figure S3. Comparison between modeled O_3 concentrations and *in-situ* measurements across distinct regions: EAC4 in China (a), Europe (b), and the US (c), as well as CMAQ in the US (d). In these boxplots, the central horizontal line represent the mean concentration, while the triangles symbolize the median value.

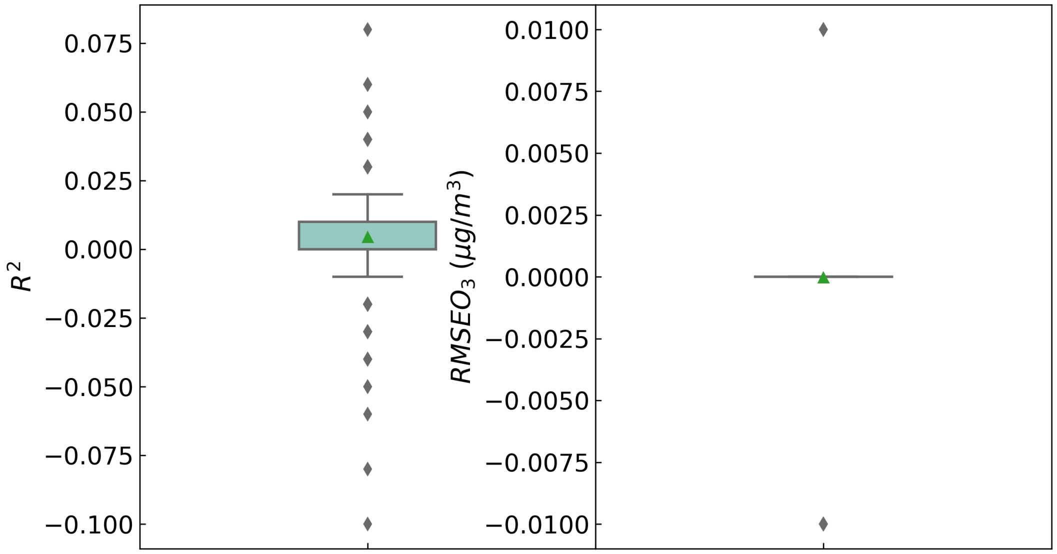

Figure S4. Differences in the accuracy of surface O_3 estimation with and without satellite columns, represented by the determination coefficient (R^2) (a) and the root mean squared error (RMSE) (b). In these boxplots, the central horizontal line represent the mean concentration, while the triangles symbolize the median value.
